# Machine Learning-Based Resource Management in Fog Computing: A Systematic Literature Review

**DOI:** 10.3390/s25030687

**Published:** 2025-01-23

**Authors:** Fahim Ullah Khan, Ibrar Ali Shah, Sadaqat Jan, Shabir Ahmad, Taegkeun Whangbo

**Affiliations:** 1 Department of Computer Software Engineering, University of Engineering and Technology, Mardan 23200, Pakistan; fahim@uetmardan.edu.pk (F.U.K.); ibrar@uetmardan.edu.pk (I.A.S.); sadaqat@uetmardan.edu.pk (S.J.); 2 Center of Artificial Intelligence for Medical Instruments, Incheon Metropolitan City 21982, Republic of Korea; 3 Department of Computer Engineering, Gachon University, Gyeonggi-do 13120, Republic of Korea

**Keywords:** machine learning (ML), deep learning (DL), cloud computing, edge computing, Internet of Things (IoT), resource management, scalability, latency, interpretability

## Abstract

This systematic literature review analyzes machine learning (ML)-based techniques for resource management in fog computing. Utilizing the Preferred Reporting Items for Systematic Reviews and Meta-Analyses (PRISMA) protocol, this paper focuses on ML and deep learning (DL) solutions. Resource management in the fog computing domain was thoroughly analyzed by identifying the key factors and constraints. A total of 68 research papers of extended versions were finally selected and included in this study. The findings highlight a strong preference for DL in addressing resource management challenges within a fog computing paradigm, i.e., 66% of the reviewed articles leveraged DL techniques, while 34% utilized ML. Key factors such as latency, energy consumption, task scheduling, and QoS are interconnected and critical for resource management optimization. The analysis reveals that latency, energy consumption, and QoS are the prime factors addressed in the literature on ML-based fog computing resource management. Latency is the most frequently addressed parameter, investigated in 77% of the articles, followed by energy consumption and task scheduling at 44% and 33%, respectively. Furthermore, according to our evaluation, an extensive range of challenges, i.e., computational resource and latency, scalability and management, data availability and quality, and model complexity and interpretability, are addressed by employing 73, 53, 45, and 46 ML/DL techniques, respectively.

## 1. Introduction

**Figure 1:**
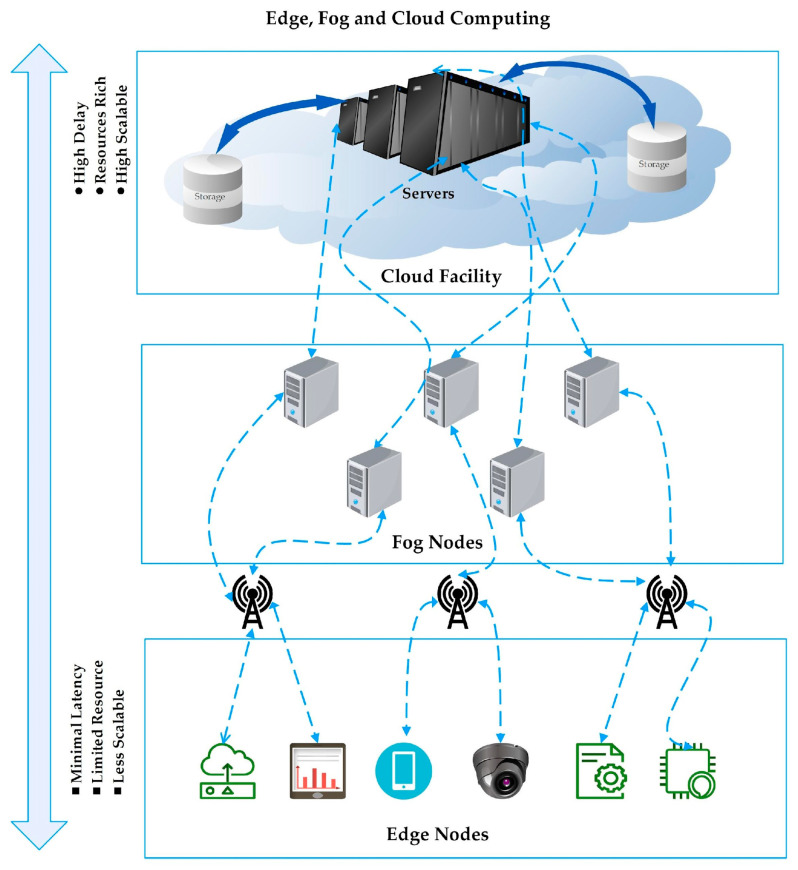
Edge–fog–cloud computing architecture.

The exponential growth of data generated by globally interconnected devices necessitates a paradigm shift in computing models. Each model offers unique services depending on its capabilities and location, as depicted in Figure 1. Among the emerging paradigms, fog computing stands out for its ability to bring computational resources closer to the IoT and end-users, thereby reducing latency and improving response times [1]. Unlike cloud computing, which offers rich resources and centralized management, fog computing operates as a decentralized approach with limited resources but greater proximity to data sources [2,3]. Key parameters of resource management in fog computing include latency, energy consumption, and computational efficiency. These parameters are critical for optimizing resource allocation and ensuring effective system performance. However, managing resources in fog computing presents significant challenges and tradeoffs, such as scalability, reliability, and cost-effectiveness. To address these challenges, ML and DL models have been extensively investigated. These models offer advanced techniques for optimizing resource management by predicting demands, allocating resources dynamically, and improving overall system efficiency [4,5]. Despite their potential, there is a lack of explicit emphasis on the key parameters that need optimization for better resource efficiency and control. This review presents a systematic literature review of ML/DL-based resource management in fog computing. Based on a thorough analysis, we identify key challenges in fog computing, essential parameters, fundamental algorithms, and the significance of critical algorithms in solving identified problems [6]. The contribution of this review includes a comprehensive analysis of these parameters and challenges, as well as effective ML/DL-based solutions for resource management in fog computing environments [7,8]. The rest of this paper is organized as follows: Section 2 presents the synthesis of the existing relevant surveys/systematic literature reviews; Section 3 presents the methodology for carrying out this research; Section 4 outlines the RQs and their significance; Section 5 presents the results, analysis, and discussions of the data obtained based on the RQs; and finally, Section 6 concludes the paper.

## 2. Related Work

ML/DL methods have been used in fog computing in numerous ways for resource management. Various surveys have been conducted that target the use of ML and DL for resource management in fog computing. Table 1 outlines the comparison of existing reviews pertaining to various ML and DL algorithms employed in previous studies to address resource management challenges in fog computing. The authors in [9] reviewed resource management challenges. In their comprehensive review [10], the authors examined resource management challenges, i.e., resource provision, load balancing, and task offloading, as well as their solutions for both fog and edge computing, and recommended future directions in the research work.

Another study [11] investigated foundational fog computing concepts and examined the application of evolutionary artificial intelligence (AI) techniques for optimizing application placement to minimize latency and maximize resource utilization. While focusing on evolutionary approaches, the study also provides detailed descriptions of relevant ML algorithms. Performance was evaluated using quality of service (QoS) and quality of experience (QoE). K.H. et al. [10] presented a review that emphasizes the growing significance of ML in fog computing. Various ML techniques were used to address challenges, i.e., resource management, latency, accuracy, and security. Though this survey explores multiple aspects of ML, i.e., application types, techniques, and datasets, it still fails to mention the performance matrix, which is necessary for the quantification and improvements to be achieved in fog computing. The survey in [7] explored the difficulties faced in the optimization of resource management in fog computing due to limited resources and dynamic workloads. The authors discussed various ML, DL, and reinforcement learning (RL) methods and proposed a taxonomy to categorize these approaches. Another survey [11] focused on deep reinforcement learning (DRL) for the data analysis of IoT in fog computing. This survey argued that employing DL in fog computing has improved its performance. The authors of [12] explored how fog computing can tackle bandwidth limitations due to massive data generated by billions of IoT. The performance matrices mentioned in this article are resource management, security, latency, and energy efficiency. A review article [13] examined resource management in fog computing with a focus on DRL. The authors argued that DRL performs well as compared to heuristic approaches and graph theory. The performance metrics mentioned in the article are QoS and QoE. Another article [14] focused on ML and how it can address issues in delay-sensitive applications. It revealed that fog applications have been classified for scheduling based on their QoS requirements to better handle them and provided future research directions on the use of ML-based task offloading in heterogeneous fog environments. However, the survey explored limited ML algorithms, i.e., decision tree and K-mean. Another review article [15] focused on how RL and DRL approaches are used to improve task offloading from resource-limited devices. DRL and RL facilitate offloading decision-making, and the former is used for large-scale networks. It categorized DRL and RL for fog offloading into three types, i.e., policy-based, value-based, and hybrid-based, and the performance metrics under consideration were latency and power consumption. However, it did not mention specific details about RL/DRL algorithms used and did not elaborate on real-world implementation challenges. In [16], RL was implemented to resolve problems of resource management in fog computing. The study established that RL offers solutions for resource allocation in fog computing amid high request rates and limitations of fog nodes. Moreover, it explored RL, which was applied to three key areas of fog computing, i.e., resource sharing, task offloading, and task scheduling. It highlighted the challenges faced by RL in dynamic environments but paid limited attention to RL algorithms and performance metrics. Another survey [17] underscored ML and DL for resource allocation in fog computing. It highlighted DRL as a promising approach for resource allocation in fog computing due to the capability it has in terms of optimization of data flow. Furthermore, it focused on latency, cost, and energy efficiency but did not explore a wide range of metrics. The authors of [18] examined resource management challenges in resource limit fog computing. They revealed six performance metrics, i.e., scheduling, load balancing, task offloading, application placement, provisioning, and latency. Their analysis showed that time response was the most evaluated performance metric. They used heuristic approaches for metrics management. A previous systematic survey [19] focused on task scheduling in fog computing. It explored various task scheduling techniques, which were categorized into four types, i.e., ML-based, heuristic-based, meta-heuristic, and deterministic. In [20], the authors reviewed emerging technologies in edge–fog computing, focusing on how these architectures address resource management issues inherent in deploying data-intensive AI/ML tasks. The efficiency gained from processing data closer to the source, minimizing latency and bandwidth constraints, is vital for the effective execution of algorithms like image recognition. However, issues such as resource allocation, management, and scalability in edge–fog deployments remain critical and are key areas of ongoing research. This paper will examine how various technologies are addressing these challenges to enable the widespread and efficient use of intelligent applications powered by ML and deep learning algorithms in edge–fog computing environments.

## 3. Methodology

**Figure 2:**
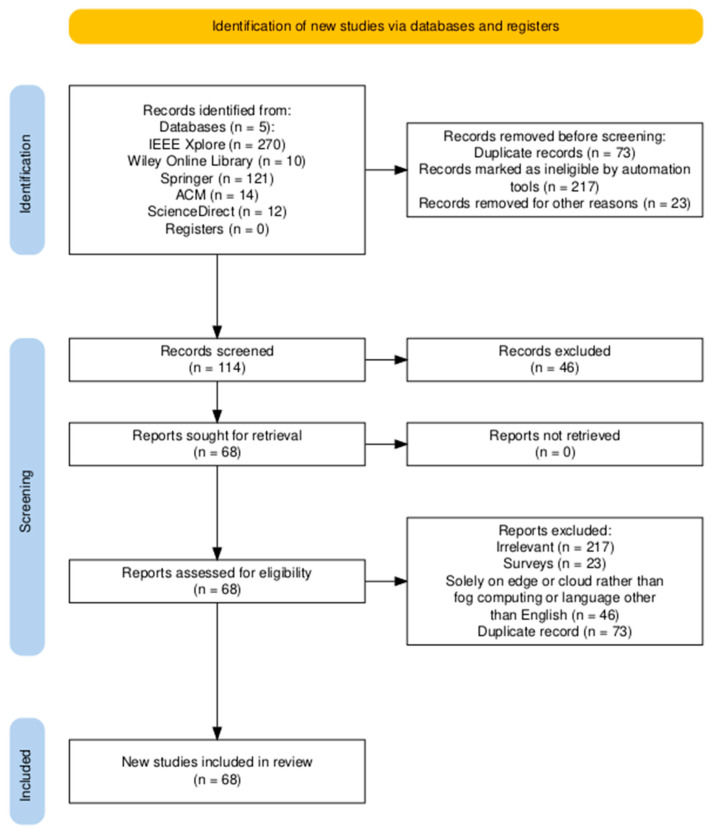
PRISMA flowchart for the article selection process for SLR.

This paper presents a systematic literature review (SLR) of ML/DL approaches for resource management in fog computing using the Preferred Reporting Items for Systematic Reviews and Meta-Analyses (PRISMA) [21]. Moreover, the protocol for this study was registered with the Open Science Framework (OSF) Registries (available publicly at https://osf.io/gjmep (accessed on 19 January 2025)). Figure 2 illustrates the PRISMA-based selection process; the relevant records were identified from databases, and ineligible and duplicate records were removed. The screening process filters were used to identify studies meeting the initial criteria, and the inclusion process led to the selection of articles for in-depth reading.

### 3.1. Identification

For conducting a scientific literature review, selecting a database is crucial for the quality and impact of the results [22]. The review process began by systematically searching five major databases (i.e., IEEE Xplore, SpringerLink, Wiley Online Library, ACM Digital Library, and ScienceDirect) that have been used to extract relevant research articles on resource management in fog computing using ML/DL techniques.

We used the keywords “resource management in fog computing” OR “Resource management in fog computing using machine learning” OR “resource management in fog computing using ML” OR “resource management in fog computing using deep learning” OR “resource management using DL” OR “resource management using deep reinforcement learning in fog computing” OR “resource management in fog computing using DRL” OR “resource management in fog computing using neuro-fuzzy” OR “resource management in fog computing using neural networks”. The time frame for this survey was from 2019 to 2024, and the publications before this time frame were not included in this survey. A comprehensive search across the designated databases yielded 279 research articles from IEEE Xplore, 10 from Wiley Online Library, 121 from Springer, and 12 from ScienceDirect. This initial pool of articles then underwent a thorough filtering process to ensure relevance and remove redundancies. This included the removal of 73 duplicate entries, 217 articles that did not feature ML and DL methodologies, and 23 surveys, resulting in a highly refined set of studies for further analysis, which is shown in Figure 2.

### 3.2. Screening

At this stage, we filtered 114 articles that were carefully screened for relevance and eligibility. In total, 46 research papers that focused on resource management and were solely on edge computing, cloud computing, or both, rather than fog computing were excluded. Having narrowed down the initial pool, 114 articles were carefully screened for relevance and eligibility. This involved a detailed assessment of each article’s core contributions to ensure they aligned with the specific scope of this literature review. The screening led to the exclusion of 46 research papers that addressed resource management in the broader context of edge computing, cloud computing, or a hybrid of both, but did not focus on fog computing. This step was crucial to refine the dataset and focus solely on publications directly pertinent to the resource management strategies within the fog paradigm and studies using DL or ML. This rigorous approach allowed us to prioritize papers that were most relevant to the research goals and objectives.

### 3.3. Inclusion

A total of 68 research papers of extended versions were finally included in this study.

### 3.4. Motivation

In recent years, instead of conventional approaches, researchers have applied ML and DL in the field of fog computing. These approaches provide diverse solutions to better manage resources, including energy consumption, cost, latency, offloading, load balancing, and other factors that affect the overall performance of fog computing. Therefore, in this work, we explored various ML and DL approaches utilized in fog computing and their impact on resource management.

To obtain better results and satisfactory information, it is paramount to draft meticulous RQs. This helps researchers gain thorough insights into the intended focus, which helps in the synthesis and identification of promising directions for further investigations. In this SLR, four RQs, as shown in Table 2, were considered. The RQs were generated based on their relevance and significance, which are outlined in Table 2.

## 4. Results and Discussion

### 4.1. RQ No. 1 What Are the State-of-the-Art ML and DL Algorithms Leveraging Resource Management in Fog Computing?

**Figure 3:**
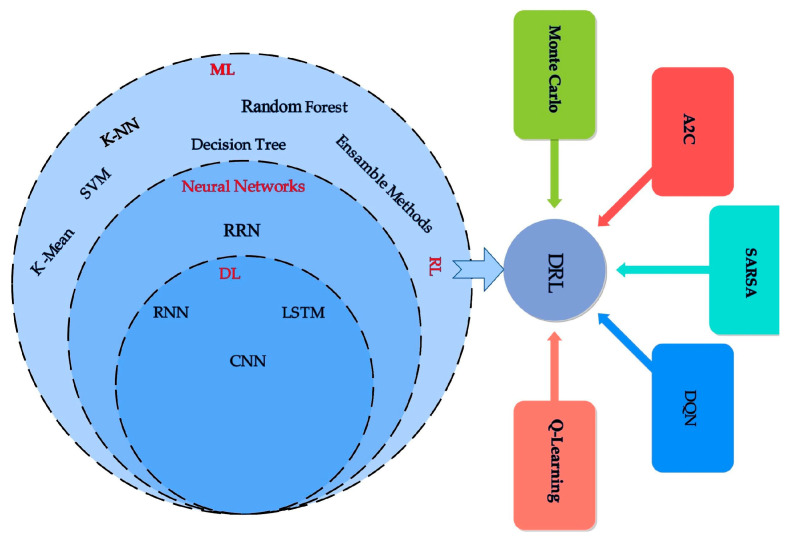
ML algorithms for fog computing applications.

It is essential to add intelligence capabilities to fog facilities to improve resource management, which in turn optimizes the operations across fog computing [23]. ML and DL have been used extensively to manage resources in a variety of real-time applications in fog computing such as healthcare [24], industrial projects [25], and vehicular fog networks [26]. This SLR encompasses state-of-the-art ML/DL techniques used to manage resources in fog computing. We selected 68 research articles for this SLR where ML/DL techniques have been used. Figure 3 categorizes various ML algorithms on resource management in fog computing into three main types, i.e., ML models comprising k-nearest neighbors (KNN), linear regression (LR), random forest, decision tree, and support vector machine (SVM); RL divided into advanced RL (e.g., multi-objective RL (MORL)); and DL consisting of convolutional neural networks (CNNs), long short-term memory (LSTM), and recurrent networks.

**Figure 4:**
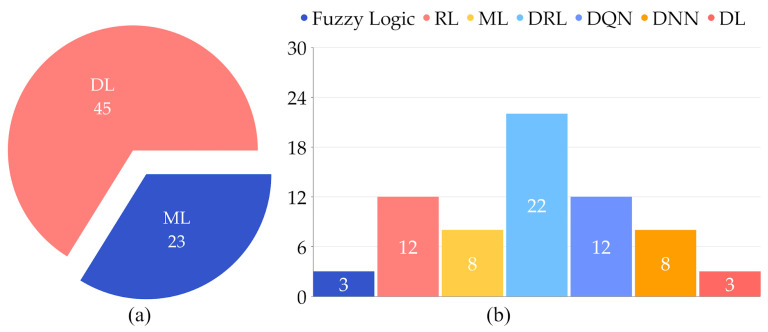
(**a**) ML/DL algorithms used in fog computing; (**b**) algorithm frequency in fog computing resource management.

Figure 4a shows 23 research papers that have addressed the issue of resource management using ML techniques, while DL algorithms have been used in 45 articles. Figure 4b shows the frequency of various algorithm usage across the same set of papers; the DRL algorithm is applied 22 times, while the deep Q-network (DQN) and RL; deep neural network (DNN); and ML, DL, and fuzzy logic are used 12 times, 8 times and 3 times each, respectively.

Table 3 represents a diverse array of algorithms used for resource management in fog computing, which varies from traditional ML models, i.e., decision tree, SVM, and random forest, to advanced techniques such as DRL and its variants like DQN, double deep Q-network (DDQN), deep deterministic policy gradient (DDPG), and deep recurrent Q-network (DRQN). Traditional ML models, such as decision trees and random forests, emerge as robust options for tasks like optimal node selection, demand prediction, and task distribution. Decision trees offer a straightforward approach but are susceptible to overfitting with complex data, whereas random forest provides greater accuracy and robustness at the high cost of increased computational intensity. The frequency column indicates that ML, fuzzy logic, DRL, and DL have been applied with a frequency of 7, 4, 41, and 13 times, respectively, in 68 research papers, providing insight into their prevalence and application in the literature. In a research study by A. Amzil et al. [27], random forest achieved the highest training accuracy in the classification of heart disease samples. Naïve Bayes is simple and efficient in probabilistic prediction and anomaly detection, faces challenges with complex dependencies, and highlights the tradeoff between model complexity and computational efficiency. Its efficient ability to predict task success makes it a powerful tool for task offloading and the optimization of resources in complex and dynamic fog environments. Mohammad S. et al. [28] used the Naïve Bayes Classifier (NBC) for classification challenges. For adaptive workload management and proximity-based load adjustment, KNN can adapt to local variation. However, it may incur high computational costs. SVM proves effective in handling large datasets and nonlinear relationships and maximizes utilization, though crucial for allocating nodes and tasks based on intricate feature vectors. SVM is best at finding hyperplanes that divide classes, minimizing overfitting and structural risk. SVM is used for both classification and regression problems. In managing resources, SVM can classify different types of tasks and predict potential resource shortages, thereby optimizing resource distribution across fog computing. Mohammad F. et al. [29] used SVM to handle nonlinear data by mapping to a higher dimensional space, which enables more sophisticated data classification through complex boundaries. These algorithms underline the importance of balancing computational efficiency with adaptive decision-making in fog computing. RL techniques, i.e., Q-learning, state–action–reward–state–action (SARSA) and their variant enable adaptive resource allocation and real-time decision-making. Q-learning adapts to a dynamic environment but is slow in convergence while, on the other hand, SARSA has a better response to immediate reward but may struggle with delayed rewards. A deep Q-learning-based offloading technique was proposed in [30], which optimizes resource allocation in vehicular fog computing. The Q-learning agent learns about the current network state (i.e., energy level of RSUs and computational latency ratio of fog servers) and makes optimal decisions about offloading of tasks. SARSA is an on-policy RL algorithm that learns policy by iteratively updating the action value estimates, which are based on the sequence of states, actions, rewards, and subsequent actions taken by the agent. It is used to train Markov decision policy (MDP). In fog computing resource management, it is used for task offloading, resource allocation, and energy management. Almuthanna N. et al. [31] proposed a solution for effective resource allocation in a fog computing environment. They used RL methods, specifically SARSA, which makes intelligent decisions for resource allocation and task offloading. DRL, DQN, DDQN, and DDPG have been noted for their ability to optimize complex policies and manage continuous actions efficiently, though the cost of computational demands is high. Fuzzy logic and neuro-fuzzy systems manage uncertainties in fog environments and enable the system for robust decision-making based on imprecise information and adaptive learning capabilities. These models require expert rule definition and are complex to implement but provide valuable insights into managing uncertain resource demands effectively. NNs, i.e., CCNs and graph convolutional neural networks (GCNNs), play a critical role in the real-time analysis of images in sensor data, network topology analysis, and graph-based resource allocation. CNNs are particularly efficient for spatial data process tasks, making them suitable for real-time decision-making in fog computing. Fan. et al. [32] developed a prediction model for patient no-shows in online outpatient appointments. ML algorithms, including bagging, random forest and boosting, were employed, achieving highest results. The potential of these models is that they improve operational efficiency in healthcare by enabling targeted intervention and better planning.

DL stands out with perfect scores, indicating its extensive and effective use in resource management and addressing issues. At the same time, RL and ML follow closely with high scores, solving the challenges in fog computing.

**Figure 5:**
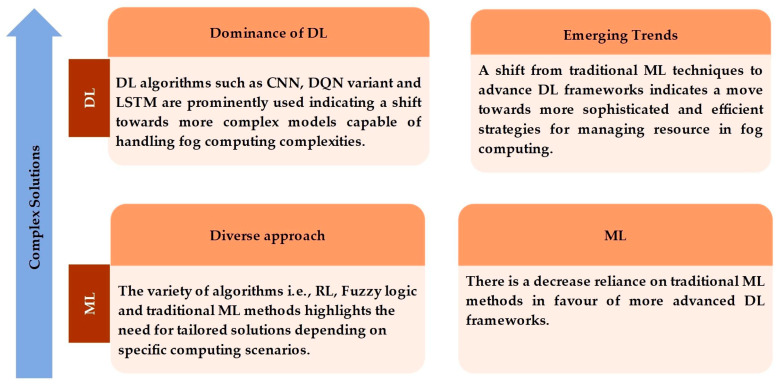
A comparative analysis of DL and ML approaches for complex solutions.

Figure 5 compares two main categories, ML and DL, in the context of complex solutions. DL algorithms are capable of handling more complicated models and computation complexities. There is a transition from traditional ML techniques to advanced frameworks, which indicates a move toward more sophisticated and efficient resource use in fog computing. This comparison underscores the evolving landscape of ML, where DL is increasingly favored by its ability to handle complex tasks and scenarios.

The implications of resource management findings in fog computing are multi-faceted. Firstly, the predominance of DL algorithms in research articles suggests a significant shift toward more complex models that can handle the complexities of the fog computing environment. This could indicate a trend where future research and applications might increasingly rely on DL for more efficient and accurate resource management. Secondly, the varied use of algorithms, i.e., RL, FL, and DQN, reflects a diverse approach to problem-solving in the field, pointing to the possibility that there is no one-size-fits-all solution and that different scenarios may require different algorithms. Finally, the relatively lower usage of traditional ML algorithms implies that these methods are becoming less favored or that they are being integrated into more advanced DL frameworks.

### 4.2. RQ No. 2 What ML/DL Algorithms Are Used for Resource Management in Fog Computing?

**Figure 6:**
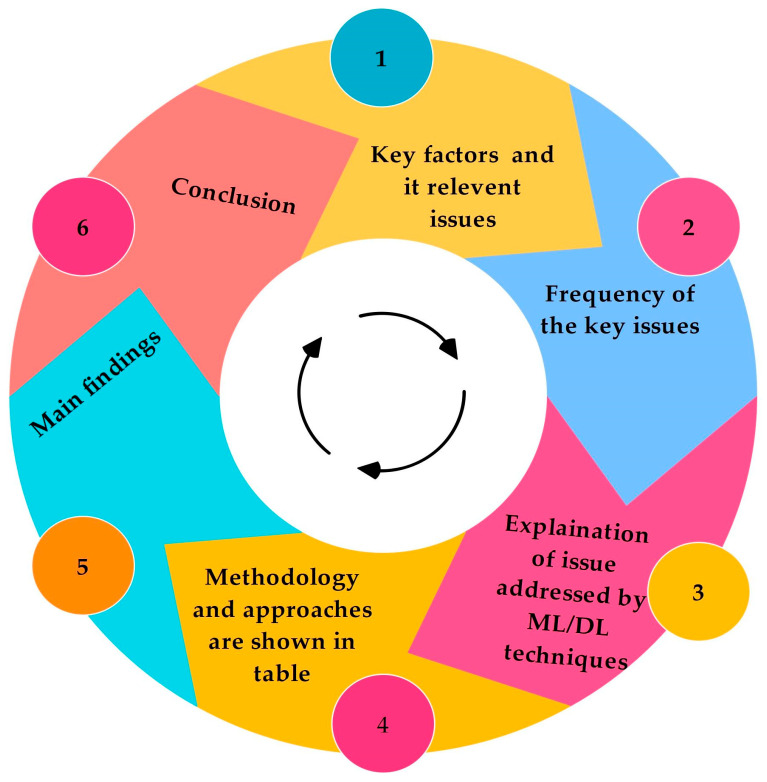
A visual guide to exploring key factors, issues, methodologies, and findings for RQ No. 2.

Figure 6 shows the road map of RQ No. 2. In this section, first, the key factors and their relevant issues are highlighted. Then, we explain how often the problems are identified in existing research. In the next section, the focus is on how ML/DL addresses these issues, followed by a table where the algorithms addressing these issues are shown. Key findings for each key factor are presented in figures, followed by a conclusion.

Real-time applications in fog computing, due to their dynamic nature of workloads, compete for scarce resources, making matters more complicated, and they raise issues like latency, power consumption, load balancing task migration, etc., which remain a concern in the evolving computing paradigm. The effective management of resources in fog computing is thus recognized as a critical challenge that demands intelligent solutions to enhance performance metrics and address issues.

**Figure 7:**
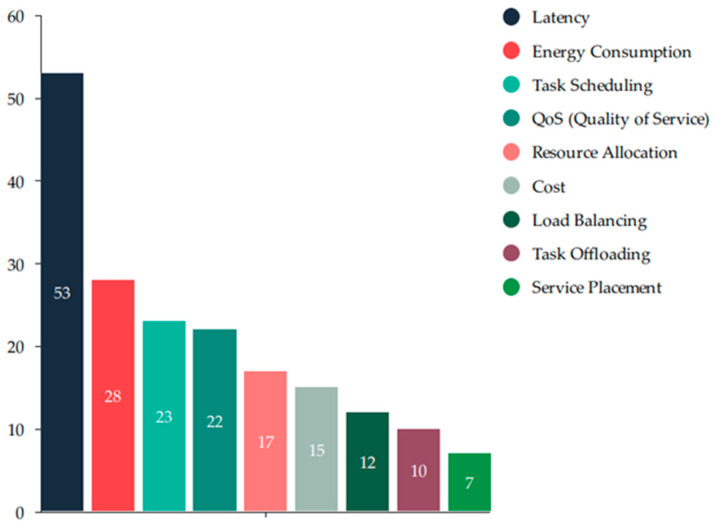
Number of articles addressing key parameters.

Figure 7 shows the frequency of issues addressed in fog computing to enhance resource management. The key issues include latency, which is most frequently discussed, with a count of 53 articles. Energy consumption is the second most frequent issue, addressed in 28 research articles, followed by task scheduling, addressed in 23 research articles. QoS appeared in 22 research articles, resource allocation in 17, cost in 15, load balancing in 12, task offloading in 10, and service placement in 7. These issues are interconnected, and addressing one affects the other. We will explore the innovative solutions provided by ML/DL algorithms.

#### 4.2.1. ML/DL Algorithms Used to Manage Latency and Their Relevant Issues in Fog Computing

Latency refers to the time taken by a request initiated by a device and data that travel to fog facilities, which are then processed and transmitted back to the device. Addressing latency is paramount in a fog environment to achieve the core functionalities of fog computing, i.e., real-time responsiveness, efficient resource utilization, and the overall stability of the system. Fog computing reduces latency by bringing computing facilities to the edge of the network, reducing network congestion and delays. Fog computing reduces the delay of edge applications in several ways. The offloading process reduces the volume of data sent to the cloud facilities, thus reducing delays. Instead of sending data to the cloud facilities, they can be processed using fog computing. Fog computing can make real-time analysis and decision-making by processing data closer to the edge. It benefits real-time applications, i.e., autonomous vehicles, health monitoring services, industrial automation, and augmented reality where prompt responses are required. Table 4 represents primary ML/DL techniques that address latency and its relevant issues with a brief explanation, mentioning its contribution and how it helps to reduce latency. Some of the methods are briefly discussed here. In their research, N.T-Pouya et al. [33] stated that the Q-learning technique is used to reduce latency by managing load balancing. This method improves the overall performance of the network in terms of run time network utilization and load distribution. The applied approach enables the system to adapt to the changing workload and make intelligent decisions to manage it. In ref. [34], a modular neural framework (BMNF) was used to decrease delay by improving the communication process and efficient utilization of fog nodes. The decrease in delay was achieved through the following mathematical model:

(1) $$delay=T^{*}-\sum_{i=1}^{n}{delay}_{i}$$

where $T^{*}\;$ is the time taken to broadcast the packets, and the delay is the difference between $T^{*}$ and the average delay of all the packets. In ref. [27], ML algorithms were used for scalable low-latency health systems. The most effective algorithm was random forest, which gave the best results. The equation below determines the total response in the fog computing system and is used to analyze and optimize its performance.

(2) $$T_{fog}=\frac{1}{N}\sum_{i=1}^{N}\frac{E_{i}^{FC}}{TH_{i}^{FC}}$$

where $E_{i}^{FC}$ is the mean number of items, and $TH_{i}^{FC}$ is the throughput of the *i*-th fog computing node. In [35], the DRL algorithm was used to handle latency in edge and fog environments. The author achieved a 60% improvement in response time by using the proposed algorithm. The total response time $\omega \left(\varphi \right)S_{i}$ is equal to the sum of the ready time and the processing time and can be mathematically expressed in Equation (3).

(3) $$\omega \left(\varphi \right)S_{i}=\omega \left(\tau \right){rS}_{i}+\omega ({proc)S}_{i}$$

where $\omega \left(\tau \right){rS}_{i}$ is the maximum time for data to arrive at the server for the task, and $\omega ({proc)S}_{i}$ is the time taken by the assigned server to process the task. In [36], a feed-forward neural network was used, which is able to predict the optimal fog node for a vehicle to offload its applications at a given location and time. A dual-stacked recurrent neural network (RNN) with LSTM cells was deployed to predict latency associated with service requests. In ref. [37], DRL was used to find the best location for IoT service in terms of cost, resource allocation, latency, and power consumption, which resulted in minimizing average power, latency, and cost while balancing resource allocation. DNN involves the estimation of value functions, which make the system capable of adapting to dynamic conditions and learn optimal strategies over time. The response time of the system is denoted by Equation (4) for latency, where *T* is total response time, $U_{i\;}$ is upload delay (propagation delay), $T_{i}$ is transferring delay (transmission delay), and P is the expected processing delay (mean execution time).

(4) $$T=U_{i\;}+T_{i}+P$$

In ref. [38], a federated multi-agent DRL was used to optimize task offloading in the highly dynamic V2X computing environment. In ref. [39], a DRL-based A2C algorithm was used to jointly optimize resource allocation and task offloading, which reduces latency. In ref. [30], a *FedDOVe* was used, i.e., DRL for decision-making and federated learning for distributed model tainting. This resulted in reduced energy consumption at RSUs, balanced computation load across fog servers, and optimized task offloading decisions, leading to reduced latency.

DRL and DL algorithms are prevalent methods for addressing various challenges in fog computing, demonstrating their effectiveness for complex decision-making and optimization. Multi-agent DRL has been used to address end-to-end delay, highlighting the importance of coordination among the fog nodes.

**Figure 8:**
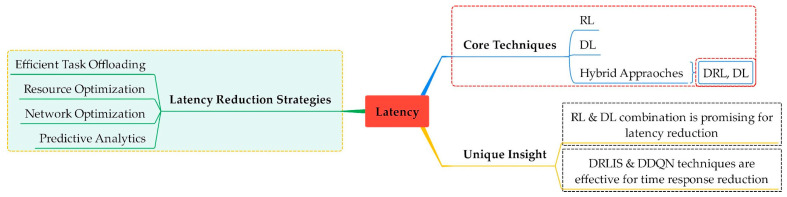
Optimizing fog computing performance: key latency reduction strategies. This mind map outlines the essential strategies and algorithms for reducing latency in fog computing environments, ensuring efficient and responsive applications.

Figure 8 shows latency as the core issue that has been addressed. Efficient task offloading, resource optimization, network optimization, and predictive analysis are the critical methods used to tackle latency.

#### 4.2.2. ML/DL Algorithms Used to Task Offloading and Its Relevant Issues in Fog Computing

Table 5 shows various advanced techniques used in fog computing for optimizing task offloading, task scheduling, and resource allocation. Algorithms, such as DRL and its variants, i.e., DRLIS, DQN, and DDQN, are prominently featured, which influences adaptive decision-making to minimize execution cost, load imbalances, and response time. FL, when combined with DQN, introduces novel approaches for efficient resource allocation and orchestration decisions based on complex factors. NNs, including CNNs and RNNs, handle workload prediction and proactive network management.

DRL, ML, and DL are the core techniques that have been used to optimize offloading. DRL and DL algorithms are prevalent methods for addressing various challenges in fog computing, demonstrating their effectiveness for complex decision-making and optimization tasks. Multi-agent DRL effectively addresses end-to-end delay by fostering coordination among fog nodes. Hybrid approaches such as DRL and SVR can enhance performance and challenge multiple addresses.

**Figure 9:**
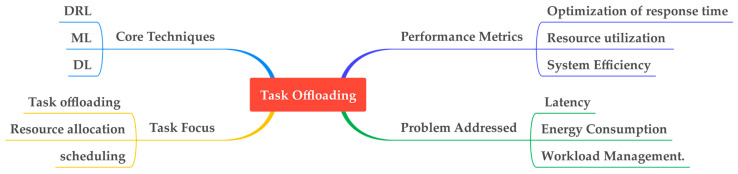
Task offloading in fog computing and strategies for minimizing latency, cost, and energy consumption.

Figure 9 shows task offloading in fog computing, highlighting the core techniques and performance metrics involved. It illustrates how advanced ML techniques can optimize task offloading, contributing to enhanced system efficiency across various performance metrics.

#### 4.2.3. ML/DL Algorithms Used for Resource Utilization and Their Relevant Issues in Fog Computing

Effective resource utilization is essential for effective fog computing services [66]. Resource utilization is the efficient allocation and management of computational resources across fog nodes to meet the demands of applications’ requested services. It optimizes resource usage, maximizing performance and ensuring high service quality. The key aspects of resource utilization are load balancing, energy efficiency, cost optimization, and QoS.

Table 6 highlights various advanced techniques employed in fog computing to optimize resource allocation and management. These techniques, including random forest, decision trees, SVM, DRL, and its variants such as DDQN, Q-learning, and DQN, are used to dynamically balance workloads across fog nodes. These techniques also allocate resources based on real-time demand and network conditions, thus efficiently utilizing available resources. NN architecture, including CNN and feed-forward network RNN, plays a crucial role in predicting source demands in data and can dynamically adjust resource allocation to minimize idle resources. FL and neuro-fuzzy offer adaptive resource management and can handle uncertainties and variations in resource demand and make decisions to ensure efficient allocation in complex environments.

DL techniques such as DNN and A2C maximize source utilization by learning optimal policies and adjusting resource allocation dynamically to match varying workloads. These systems enhance overall system efficiency and throughput and collectively contribute to improving the efficiency and performance of fog computing across diverse applications. A previous study [67] focused on the optimization of the placement of microservices in a fog computing environment to improve resource utilization and system performance. A DRL-based approach was used to orchestrate interconnected microservices across computing facilities. This approach makes optimal placement decisions based on resource constraints and system dynamics. The serverless architecture enables scalable and flexible resource management in fog computing facilities. This approach can achieve effective microservice composition, which results in improved resource utilization and system performance. In another study [37], a DRL approach was used to address the resource allocation problem in fog computing.

DNN estimates the value functions and improves adaptability to diverse network conditions, while DRL enables the learning of optimal resource allocation strategies based on system interaction. In [68], DRL was used to efficiently place services in a fog computing environment to meet the increasing demands of IoT applications. It optimizes service placement according to resource availability, the diverse response of the network, and user demands. It uses DRL-based intelligent fog and service placement (IFSP) to enhance the performance and responsiveness of fog computing systems by optimizing service placement. In [69], a joint renewable energy cooperation and resource allocation scheme was proposed. DQN and Q-learning were used to optimize power allocation and resource allocation.

Effective resource utilization is a critical aspect of fog computing, which ensures optimal performance and high-quality service. By using advanced ML/DL learning techniques, researchers have made significant strides in optimizing resource allocation and management. Techniques such as random forest, SVM, decision tree, DRL, and its variants, along with neural networks and FL, have demonstrated their effectiveness in addressing dynamic workloads, uncertainty, and resource constraints. These approaches enable dynamic workflow balance, efficient resource allocation, and adaptive resource management, which results in the ultimate enhancement of system efficiency and overall performance and throughput. As fog computing continues to evolve, the application of these techniques will be essential for meeting the growing demands of resource-intensive applications and ensuring the success of future fog-based solutions.

**Table 6:** Overview of ML/DL algorithms used to address resource utilization and its related issues.

Techniques	Explanation	Issue Addressed	Refs.
Random Forest	ML techniques such as random forest, decision tree, and SVM can predict the task’s nature and optimize resource allocation based on previous data and patterns, which leads to efficient utilization of available resources.	Efficient resource allocation	[27,28,54]
Decision Tree			
SVM			
DRL	These RL algorithms can optimize resource allocation by adopting policies that can balance workload across fog nodes, make the distribution of resources fair, and allocate them based on real-time demand and network conditions, which leads to efficient resource utilization.	Optimal resource allocation	[26,30,35,37,38,40,44,53,65,67,68]
DDQN			
Q-learning			
DQN			
CNN	Neural network architectures like CNNs, RNNs, and feed-forward networks optimize resource utilization by learning patterns in data, predicting resource demands, and dynamically adjusting resource allocation to improve efficiency in processing tasks and minimizing idle resources.	Resource utilization	[25,36,43,58,70]
RNN			
Fuzzy Logic	FL and neuro-fuzzy systems can handle uncertainties and variations in resource demands. So, these systems can adaptively manage the resources across fog computing environments. Fuzzy sets and rules can make decisions that optimize resource utilization and ensure efficient allocation in complex environments.	Adaptive resource management	[47,55,56,59,62]
Neuro-Fuzzy			
Fuzzy Q-learning			
DL	DL techniques such as DNN and A2C maximize resource utilization by learning optimal policies that help minimize idle resources, adjust to dynamic resource allocation to match varying workload demands, and improve throughput, which leads to enhanced overall efficiency.	Maximizing resource utilization	[34,37,39,69,71]
DNN			
A2C			

**Figure 10:**
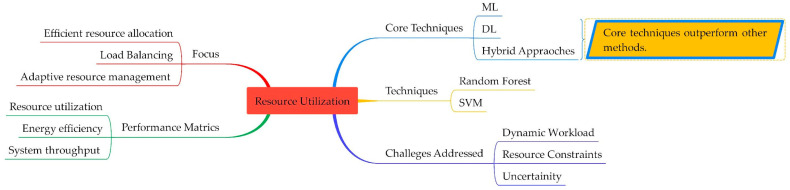
Resource allocation in fog computing using ML, DL, and hybrid approaches.

Figure 10 highlights the multi-faceted approach required to optimize resource allocation effectively.

#### 4.2.4. ML/DL Algorithms Used to Power Consumption and Their Relevant Issues in Fog Computing

Power consumption is a significant concern in fog computing due to the diverse nature of a large number of devices and the distributed nature of the infrastructure [72]. Power consumption in fog computing can be managed through efficient workload handling, resource utilization, and power management strategies [73]. Table 7 presents the techniques used to address issues to reduce power consumption with a brief explanation. Some of the research articles are mentioned here. In [25], a CNN-based system was deployed, which enables the system to perform dynamic prognosis and optimizes the machining process. A three-layer architecture is in place, i.e., a terminal layer that collects power signals for machines; a fog layer that processes data and runs trained CNN for fault detection; and a cloud layer that can handle intensive computing, i.e., CNN training and the re-optimization of CNN-based prognosis. This system improves energy and production efficiency in real-world manufacturing environment. In [45], the authors proposed that DL-JODRA algorithms achieve optimal offloading decisions and effective resource allocation policies, significantly reducing network issues such as delay and energy consumption. A clipped double deep Q-learning (DDQLS) algorithm [53] minimizes the average energy consumption by accelerating task execution time and makespan. It enables the optimization of virtual machine (VM) selection based on resource availability and power consumption, which affects the overall energy efficiency of the system. In [43], the DNN algorithm was used, which is capable of optimizing energy consumption by intelligently managing task offloading with different task loads from various users across heterogeneous fog nodes. Another study [54] used ML for resource management and proportionate energy consumption with computer utilization. It incorporates various energy consumption factors, i.e., sensors, which consider idle and active power states, data transmission, and task processing, which includes transmission, execution energy, bandwidth and computational cycles.

(5) $$E_{total}=E_{sensors}+E_{classification}+E_{computational}$$

Equation (5) shows that the consumption of total energy is the summation of energy consumed by sensors, energy consumed by the classification algorithm, and computational energy. A gated graph convolution network (GGCN) with CNN was employed [74] to efficiently utilize resource and energy consumption. However, energy consumption usually occurs at cloud facilities. In this research work, an RNN was used in which the energy consumption model evaluates the energy of the system by considering sensors, the energy consumed by the classification algorithm, and task computation depicted in Equation (6).

(6) $$E_{total}=E^{transimission}+E^{idle}+E^{utilization}$$

where $E^{transimission}$ is the transmission energy of the sensor to the fog layer, $E^{idle}\;$is an idle energy of the devices, $E^{utilization}$ is the load energy utilization. In [69], a hybrid power supply was used, i.e., renewable energy and grid power. Q-learning and DQN were employed for power regulation by adjusting power allocation, which was based on the network’s real-time conditions, i.e., channel quality and user demand.

**Figure 11:**
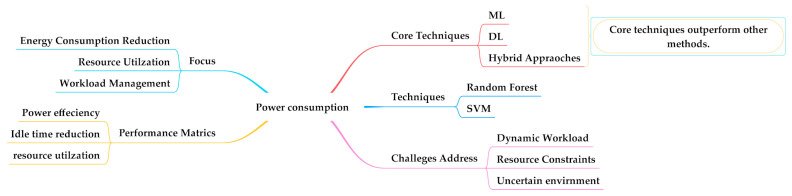
Power consumption in fog computing.

Power consumption is a critical concern in fog computing, requiring efficient management strategies to optimize energy efficiency and minimize cost. ML/DL methods have been applied to develop innovative approaches to address these challenges. Techniques such as random forest, SVM, decision trees, and various DRL algorithms have demonstrated their effectiveness in optimizing task offloading, scheduling, and resource allocation, resulting in reduced energy consumption, as depicted in Figure 11.

#### 4.2.5. ML/DL Algorithms Used to Address Service Placement and Their Relevant Issues in Fog Computing

Service placement in fog computing is to identify an optimal location for deploying services within the network. It aims to minimize latency and maximize resource utilization. A key challenge in fog computing is resource heterogeneity, such as computational power, network bandwidth, and storage capacity, which poses a challenge in finding suitable placement. Besides resource heterogeneity, dynamic workload, network topology, and fault tolerance are the challenges in fog computing; if addressed, effective service placement can significantly improve the performance of fog systems [75].

Table 8 outlines ML, DL, and RL algorithms, along with the issues they address and their explanation. In [68], DRL was applied, which enables the system to learn optimal service placement decisions over time. It helps the fog computing environment to meet the increasing demands of IoT applications. The key issues it considers while optimizing the service placement are resource availability, user demands, and dynamic changes in the network. It modifies DRL to IFSP, which helps make the system scalable and can address the challenges of a diverse environment and resource constraints.

In another study [40], DDQN and prioritized experience replay were used to optimize service placement by minimizing the time-of-service execution and managing the energy of a service node efficiently. This DRL-based approach learns optimal service placement decisions over time and can be adapted to changing conditions. Prioritized experience replay enhances the learning process, which improves system performance. In [63], an A3C algorithm was used for service placement in fog computing to optimize resource utilization and meet deadlines. A special queue was in place to manage the service requests. The incoming services were placed on available fog nodes based on resource constraints and timely execution. Another study [56] used a neuro-fuzzy approach to effectively allocate resources in a fog computing environment. It selects virtual machines based on their efficiency and resource availability. The efficiency check ensures that the selected VM is in accordance with the application resource requirements. Each task must be assigned to only one VM denoted by M. The task is denoted by T,

(7) $$\sum_{i}\sum_{j}\sum_{k}Y_{T,i,j,k}=1\;for\;all\;T$$

The total resource consumption of a task assigned to a VM cannot exceed its capacity,

(8) $$\sum \sum_{T}\left(P_{T\;}*Y_{T,i,j,k}\right)\leq Q_{i,j,k\;}for\;all\;i,j,k$$

The VM must meet the efficiency threshold; efficiency is denoted by E and the threshold by ψ

(9) $$E_{i,j,k}\geq \;\psi ,\;if\;T_{Y,i,j,k}=1$$

where $\left(Y_{T,i,j,k}\right)$ represents the assignment of task T at VM M and at indices i,j,k. Similarly, $P_{T}$ represents the resource consumption of task T for all; $Q_{i,j,k\;}$ represents the capacity of VM M at indices i,j,k; $E_{i,j,k}$ represents the efficiency of VM M at indices i,j,k; and the threshold is defined by ψ.

The critical role of ML/DL algorithms in optimizing resource allocation and reducing costs in fog computing environments. Techniques such as DRL, FL, and Q-learning are instrumental in automating resource provisioning, enhancing system performance, and mitigating operational expenses. These algorithms successfully address challenges like dynamic workload management, uncertain information handling, and efficient resource utilization. Besides optimizing service placement and load balancing, they also minimize energy consumption and communication costs. Moreover, the frameworks discussed emphasize the importance of aligning resource allocation with SLAs to ensure cost-effective operations and sustainable scalability in fog computing systems. This comprehensive approach not only enhances system efficiency but also supports adaptive decision-making, which results in fog computing, a viable solution for future IoT and edge computing applications.

**Figure 12:**
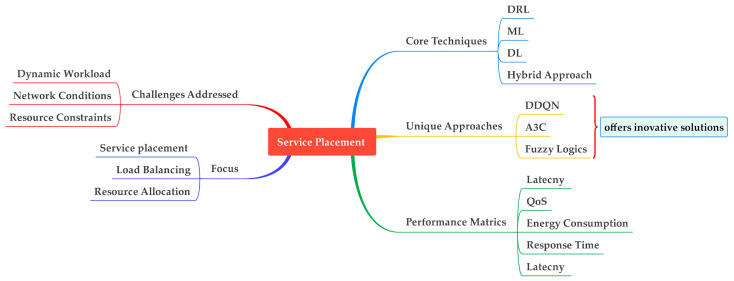
ML/DL techniques for service placement: tackling challenges and enhancing performance metrics.

Figure 12 depicts a multi-faceted approach to service placement by addressing challenges such as dynamic workloads, resource constraints, and uncertainty. To overcome these issues, various techniques are employed, including resource allocation, adaptive resource management, and load balancing. ML/DL and hybrid approaches enhance the precision and adaptability of resource management. Performance metrics such as resource utilization, system throughput, and energy efficiency are crucial for evaluating these techniques, which results in optimal resource distribution and system performance. This comprehensive framework features the complexity and interdisciplinary nature of effective resource allocation in a modern computing environment.

#### 4.2.6. ML/DL Algorithms Used to Address Cost and Their Relevant Issues in Fog Computing

Table 9 shows ML/DL algorithms that are revolutionizing cost management in fog and other paradigms by optimizing resource allocation, adopting adaptive scheduling, and reducing operational costs through these techniques. These advancements not only provide QoS and address service placement challenges but also align resource utilization with cost-saving goals. With the evolution of these technologies, they provide sustainable, improved performance and scalability of IoT applications. In [62], the process of allocating resources during the fulfillment of requests is automated in fog computing. The incorporation of FL makes the system able to handle uncertainties and imprecise information effectively. The integration of Q-learning in the system allows it to learn optimal resource allocation policies over time. Optimal resource utilization reduces idle resources, resulting in cost savings. Unnecessary resource consumption is avoided, which leads to lower energy costs. Efficient resource provisioning, on the one hand, enhances system performance and, on the other hand, reduces operational costs. This framework assesses the cost violation during resource provisioning against the expected cost defined by SLAs. The assessment is used either to scale up the system or scale it down accordingly. The F-AMLF framework [47] enables efficient resource utilization by accurately estimating computing capacity requirements, avoiding over-provisioning, and optimizing resource sharing, resulting in a cost reduction in the system. In another study [76], the authors used a predictive energy-aware scheduling framework to address explicit issues of bandwidth waste resource depletion and high operating costs, leading to cost reduction. The total execution cost is shown in Equation (10).

(10) $$TotalCost\left(TC\right)=\sum_{i=0}^{N}CPUcost\left(k\right).Y_{i,k}+\sum_{i=0}^{N}MemoryCost(k).Y_{i,k}$$

*CPUcost(k*) is task(*k*) and its potential assignment to a particular VM(*i*). *CPUcost(k)*. Y*_i_*_,*k*_ is the total CPU cost for all *N* tasks; it adds up the *CPUcost(k)* for each task *i* that is assigned to some VM *k*, and *MemoryCost(k)* is the amount of virtual machine memory for each task(*i*). Using DRL, this framework [46] addresses cost issues in fog computing by considering communication cost and resource depletion. It defines proximity in terms of communication cost, which minimizes the use of distant nodes for processing to reduce communication costs and bandwidth wastage. It integrates cost factors directly into environment dynamics and the reward function that trains the RL agent to efficiently make cost-effective scheduling decisions that result in cost reduction.

**Figure 13:**
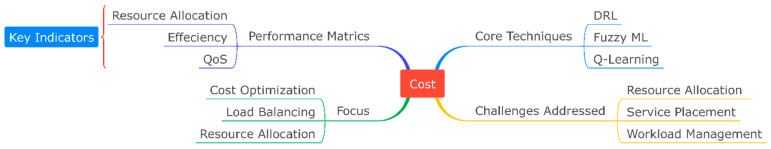
Cost optimization: utilizing DRL, fuzzy ML, and Q-learning for efficient resource management and cost optimization.

Figure 13 shows core techniques, challenges, and performance metrics. At the same time, it shows that the focus is on cost optimization, load balancing, and resource allocation. The challenges addressed are service placement, resource allocation, and workload management.

The integration of ML/DL algorithms in fog computing to address cost-related challenges reflects a strategic approach toward optimizing resource utilization and operational efficiency. Research efforts such as incorporating FL and excellent learning highlight advancement in handling uncertainties and dynamically learning optimal resource allocation policies over time. These techniques minimize not only idle resources and reduce energy costs but also enhance overall system performance by efficiently provisioning resources according to service demands. Implementing frameworks like DRL and predicting energy-aware scheduling further emphasizes the importance of aligning resource provisioning with cost-effective strategies, ensuring adherence to SLAs, and mitigating bandwidth wastages. By integrating cost factors directly into the decision-making process through DRL-based approaches, fog computing systems can achieve significant cost reduction while maintaining robust service quality and scalability.

#### 4.2.7. ML/DL Algorithms Used to Address Load Balancing and Their Relevant Issues in Fog Computing

Load balancing is a critical aspect of efficient resource utilization in fog computing. Table 10 presents how the issues are addressed using different algorithms. Algorithms such as DQN and DRLIS are pivotal for load balancing and resource utilization. It improves system reliability, enhances energy efficiency, and optimizes response time. Federated deep Q-learning (DQL) distributes workloads and boosts the system’s performance. Load balancing avoids situations where fog nodes are underloaded or overloaded, which can lead to performance issues. Load balancing is ensured in fog computing through the distribution of user requests on VMs in equal proportions to avoid overburdening or underutilization of nodes. Task scheduling is another technique that helps load balancing in fog computing, i.e., tasks are scheduled in such a way that the resources in the fog environment are efficiently distributed [77]. The DDQL-based scheduling approach [26] distributes tasks in such a way that it prevents fog nodes either from being overburdened or underutilized. Distributed decision-making improves scalability, which results in efficient load balancing. DRL addresses load balancing [34] with the scheduling algorithm DRLIS. It optimizes the response time of heterogeneous IoT applications and balances the load of processing servers. In [30], the DQL-based *FedDOVe* algorithm was used to address the issue of load balancing across the fog servers by reducing execution cost, response time, and weighted cost. In [57], the issue of load balancing was addressed through a dependency-aware collaborative vehicle edge–cloud task offloading scheme, whether the tasks were processed locally or offloaded to cloud servers.

**Figure 14:**
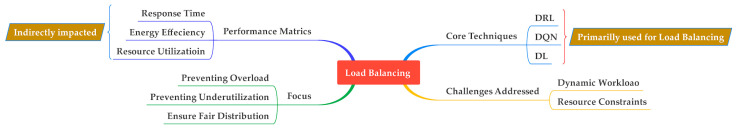
Load balancing: enhancing system performance with dynamic resource allocation strategies in fog computing.

Figure 14 represents how various factors and techniques influence load balancing. Load balancing is essential in fog computing for efficient resource utilization. Multiple algorithms, including DQN, federated DQN, DL techniques, and DRLIS, have been employed to address load-balancing challenges. These algorithms distribute tasks across fog nodes, balance workload, and optimize response time, which ensures that nodes are not overburdened or underutilized; due to this effectual management of load balancing, these techniques contribute to improved overall system performance and resource efficiency in the fog computing environment.

#### 4.2.8. ML/DL Algorithms Used to Address QoS and Their Relevant Issues in Fog Computing

Table 11 provides a comprehensive overview of how different techniques and algorithms are used to address various issues in network environments, resulting in optimal performance and efficiency. QoS in fog computing can be maintained in several ways, i.e., connectivity, delay reduction, capacity management, network aware scheduling, SLAs, and resource scheduling. These factors ensure that the applications served in the fog environment have delivered a quality service to the end-user [80].

**Figure 15:**
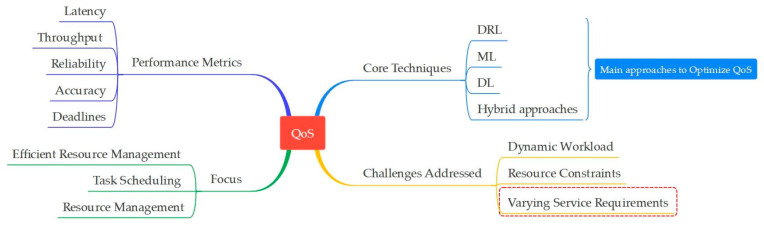
A comprehensive overview of QoS optimization in fog computing, featuring DRL, ML, and hybrid approaches.

Figure 15 highlights the key factors and techniques involved in optimizing QoS, ensuring efficient and reliable system performance. The integration of the ML and DL algorithms in fog computing demonstrates a robust approach to enhancing QoS across various critical dimensions.

DRL and its variants DQN and DDQL stand out for their effectiveness in optimizing latency, throughput, reliability, accuracy, and meeting deadlines. NNs, i.e., CNNs and RNNs, complement these efforts by predicting and reducing latency through efficient task scheduling and resource utilization. Q-learning enhances throughput by optimizing load balancing and resource allocation, thus ensuring system efficiency; these advancements not only improve service delivery but also mitigate SLA violations through precise decision-making and enhance performance metrics. By leveraging these technologies for computing environments, high-quality service can be consistently provided while adapting to dynamic network conditions and user demands, thereby enhancing overall system performance and user satisfaction.

### 4.3. RQ No. 3 What Are the Major Challenges That Have Been Addressed During Resource Management in Fog Computing Using ML/DL Techniques?

While fog computing offers a compelling solution to the limitations of traditional cloud computing by bringing computational resources closer to the edge of the networks, it carries a unique set of challenges that must be addressed to ensure optimal resource management. To address these challenges, a multidisciplinary approach is required that combines both ML and DL techniques and may take into account the domain-specific knowledge of fog computing environments. The ongoing research in the field of fog computing has resulted in the development of scalable, secure, trustworthy, and efficient ML/DL solutions, which results in effective resource management and enhanced performance of fog computing. These research efforts are necessary to extract the full potential of fog computing in various applications while addressing the challenges faced due to the heterogeneous and distributed computing environment. A holistic approach is essential to overcome these challenges; improvement in one area can impact others. Therefore, strategic planning and integrated solutions are necessary for robust, efficient, and secure fog computing systems.

Table 12 provides a comprehensive overview of the challenges faced in fog computing. Limited computational resources, including processing power, memory, and storage, significantly affect fog devices’ ability to deploy complex ML and DL models, which results in the suboptimal performance of computationally insensitive applications. Efficient model training techniques and lightweight models are essential to optimize the impact of the deployment of large datasets and pre-trained models [86]. Meeting the stringent latency requirement of real-time applications is a critical challenge in fog computing. Network delays and computational limitations can impact the overall performance and responsiveness of applications. The challenges highlighted in the table directly impact resource management in fog computing. These challenges affect application performance, while latency constraints are carefully needed to prioritize time-bond tasks. Scalability and management are crucial to handle system complexity and large-scale fog node deployments. ML/DL techniques enable distributed resource management and microservice architecture, ensuring scalability and efficient resource provisioning. Addressing node heterogeneity and providing fault tolerance through robust monitoring and resource allocation strategies like DQN, DDQN, and MORL support seamless deployment and maintenance of fog computing infrastructure. Insufficient and varied data formats usually affect model accuracy. Solutions are generating synthetic data, deploying federated learning, and utilizing data augmentation techniques to enhance data quality and model performance. Model complexity can strain computational resources and challenge performance. Optimizing models through ML/DL techniques mitigates these challenges, improving efficiency and resource utilization.

Furthermore, model transparency and reliability are critical for decision-making and pattern recognition. The dynamic adaptation of models to varying network conditions and efficient deployment strategies like DL facilitate continuous learning and value updating, optimizing performance in dynamic fog computing environments. Tailored ML/DL approaches can address these challenges, which result in enhanced efficiency, scalability, and reliability in fog computing by supporting diverse applications and maintaining operational excellence in complex computing landscapes.

In Table 13, different algorithms are shown, which demonstrates a holistic approach to addressing diverse challenges in fog computing, emphasizing efficiency, performance, and reliability. ML algorithms are primarily focused on traditional ML techniques to optimize resources and manage energy consumption effectively. RL algorithms dynamically optimize resources, reduce latency, and enhance real-time performance across distributed computing environments. DL techniques are used for complex pattern recognition, optimizing response time, and achieving significant performance improvements through innovative approaches like federated learning and multi-objective optimization.

**Figure 16:**
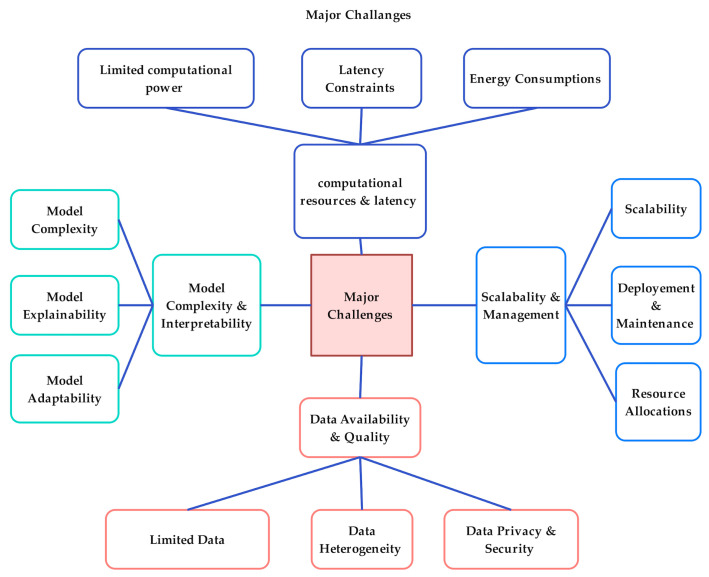
A comprehensive overview of the key challenges hindering the adoption of fog computing technologies.

Figure 16 highlights the interconnected challenges in fog and edge computing, emphasizing that solutions must address multiple dimensions simultaneously. Computational power, latency constraints, energy consumption, scalability, deployment and maintenance, and data privacy and security are all critical factors

**Figure 17:**
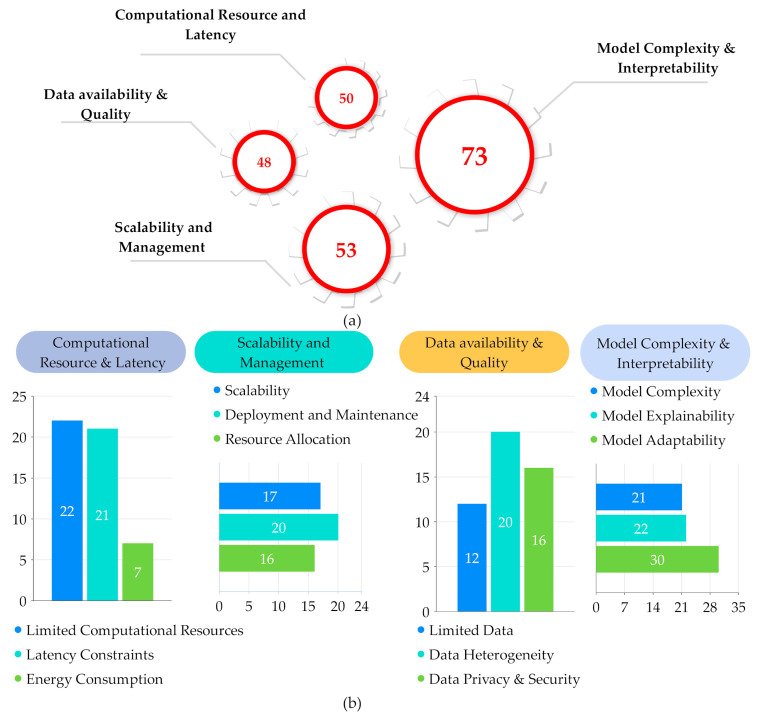
ML/DL methods used to overcome the challenges in fog computing.

Figure 17a show a significant number of ML/DL algorithms focus on addressing model complexity and interpretability, with 73 algorithms targeting this challenge. Followed by scalability and management, which is addressed by 53 algorithms, computational resource and latency with 50 algorithms and data availability and quality with 48 algorithms. In Figure 17b, the analysis of computational resource and latency indicates that 22 algorithms are designed to manage limited resources, 21 algorithms address latency constraints and 7 algorithms focus on optimizing energy consumption. For scalability and management, 17 algorithms that tackle scalability issues, 20 algorithms dedicated to deployment and maintenance and 16 algorithms for resource allocation. Model complexity, explainability and adaptability with 21 targeting complexity, 22 focusing on explainability and 30 on adaptability.

**Figure 18:**
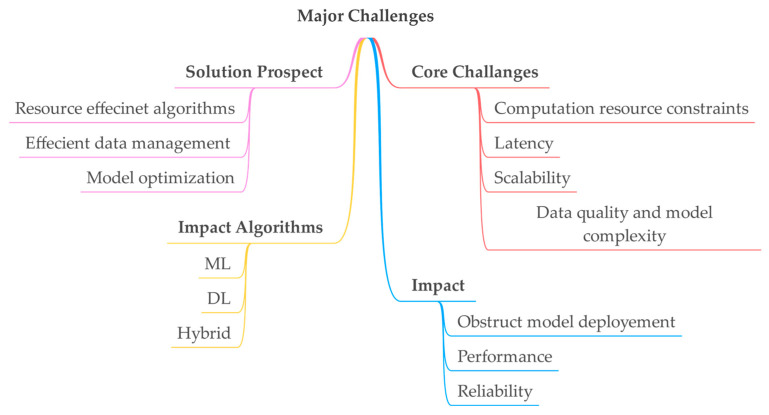
ML/DL approaches for addressing core fog computing challenges.

Figure 18 depicts the intricate challenges in fog computing, which are addressed by deploying various ML and DL algorithms. These algorithms provide efficient resource management, efficient data management, and model optimization and address the core issues such as computational resource constraints, latency scalability, data quality, and model complexity. These core challenges impact performance, reliability, and ability to deploy models effectively

### 4.4. RQ No. 4 How Do ML/DL Methods Enable Dynamic Resource Allocation and Distribution Across the Layers, i.e., Use Edge–Fog or Edge–Fog–Cloud, or Manage It on Fog?

The fair allocation of computational tasks across the three layers, i.e., edge, fog, and cloud environment, is pivotal in optimizing system performance, cost, and security. The decision of task distribution is based on several key factors, such as computational complexity, latency constraints, data volume and bandwidth, cost, security, and privacy [87]. The research in [35] leveraged dynamic resource allocation methods based on RL and genetic algorithm (GA) and aimed to address the challenges of resource allocation across the layers. A multi-layer system comprising IoT, fog, and cloud layers was used to monitor patient symptoms and collect real-time data through connected medical devices. The fog layer was involved in managing incoming requests from edge devices and distributing them across appropriate fog servers. The cloud layer was involved in overseeing data management and transfer of information between the fog layer and healthcare servers. It ensured that the patient data were offloaded to the most suitable server based on load and processing capabilities. The task allocation to cloud servers depends upon specific conditions, i.e., overload at fog servers, task complexity, resource availability, and load balancing. In [27], IoT devices collected patients’ health data and sent it to the servers for processing and classification. The data were classified as high-risk and low-risk by using SVM. The decision made on whether the application is processed on a fog node or cloud node depends upon the nature of the data classified. The high-risk data are time-critical and need to reflect a timely response, and they are processed on fog nodes. However, for in-depth analysis, the high-risk data are also sent to the cloud facility. At the same time, the low-risk data are sent to a cloud facility for processing. In [46], a DRL approach was used to place applications within fog or cloud facilities. The decision on the system task placement is influenced by three factors: current node load, node distance, and priority of the task. The task distribution criteria are defined by MODRL, where tasks involving large volumes of data, time-critical and cost-sensitive, are provided at fog nodes.

On the other hand, complex tasks are provided on cloud facilities. The proposed framework [37] outlines a dynamic resource provisioning system that involves job migration between different layers. Job migration is influenced by factors such as request deadlines, resource availability, network congestion, and load balancing. The data generated by IoT devices are sent to the fog layer for processing, where the fog layer processes the data based on available resources. If the fog layer is overloaded or the request deadline is exceeded, the job is migrated to the cloud. The DRL agent decides on the placement of a job based on the mentioned factors, either to be served on the fog layer or the cloud layer. Table 14 presents various algorithms used for resource management within multi-layer computing environments, i.e., edge, fog, and cloud architecture. The algorithms come with computational architecture, within which it operates, and the types of tasks it can handle are indicated. These algorithms vary in complexity and methodology, addressing the challenges posed by resource distribution across edge, fog, and cloud environments. The specified architecture for each algorithm indicates the computational layer involved, i.e., edge, fog, and cloud. The edge layer typically handles data generation and processing near the data source. The intermediary fog servers provide closer access to the cloud, which employs robust computational power for more extensive data processing. Some of the tasks are solely managed at the edge or fog level, while others may migrate to the cloud based on specific conditions or resource availability, making the algorithms adaptable to varying operation contexts.

**Figure 19:**
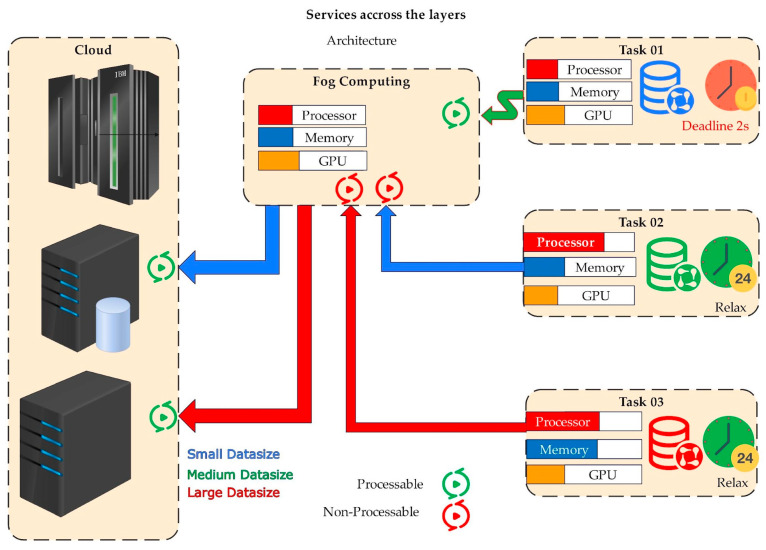
A hierarchical cloud–fog computing architecture demonstrating task offloading, resource allocation, and data processing based on data size and deadline.

Figure 19 illustrates a distributed computing architecture that employs computing to facilitate data processing closer to its source. These are designed to minimize latency and enhance response time, particularly for applications demanding real-time data processing. The fog computing layer consists of computing resources situated at the network edge near the data resources and end-users. It includes fog nodes equipped with processor memory and other resources. Tasks are created, and associated data accompany each task. The data are sent from their source IoT devices through the fog nodes. Fog nodes access the data size and computational needs for each task. If the task is processable, it is executed on the fog nodes. Otherwise, it is forwarded to the cloud for processing, and the outcome of the processing is sent back to the original data source or end-user. This architecture reduces latency, improves scalability, and enhances reliability.

## 5. Practical Implementation of ML/DL Algorithms

Table 15 depicts the practical implementation of various ML/DL algorithms that have been used in research studies pertaining to resource allocation in fog computing. The algorithms were analyzed and validated based on key metrics with real-world scenarios. As shown in Table 15, industrial monitoring and analysis were carried out in a real-world scenario where a CNN was used as the learning algorithm based on the key metrics of energy and production efficiency. This fog-based system resulted in a 29.25% increase in energy efficiency, a 16.50% rise in production efficiency, and a 70.26% decrease in bandwidth requirements, as well as a 47.02% reduction in data transfer time. In another real-world scenario, smart cities and traffic control [88] were modeled using the RL algorithm considering smart cities or IoT environments for fog computing resource management. In this study, a consistent ability to maximize task completion within deadlines was demonstrated. In another real-world scenario pertaining to vehicular fog networks, it was demonstrated that user requests were satisfied within a delay threshold, and the DRL algorithm outperformed heuristics approaches [78]. Similarly, the authors of [83] modeled vehicular networks in a fog environment using DRL as the learning algorithm. The results show that the task completion ratio was the highest and the method outperformed all other algorithms in consideration even in high vehicular density. In another real-world scenario involving a vehicular fog network [38], the federated multi-agent DRL algorithm was applied, which reduced network overhead. The results from that study show that at an arrival rate of λ = 200, the task residence time decreased by 58.6%, the end-to-end delay decreased by 57.11%, and the overall system cost decreased by 16% as compared to other studies. The FedDOVe [30] technique, which uses DRL as the learning algorithm, was used in a vehicular fog network, and substantial improvements were achieved in several key areas as compared to the existing studies. The results demonstrate a 47–49% average reduction in energy consumption, a 60–65% average improvement in load balancing, and a 60–65% average reduction in mean computation latency as compared to other existing tested models. In the healthcare domain, FogDLearner [70] was modeled as a real-world cardiac management system that uses DRL as the learning algorithm. The system achieved an accuracy of 84.91% and 82.26% precision. The recall and F1 score of the model were 91.07% and 86.44%, respectively. To demonstrate the five-layered neuro-fuzzy model effectiveness, four healthcare applications, augmented reality, patient pre-monitoring, record analysis, and billing system, were assessed based on their diverse parameters in the research study of [56], which reported improvement of 2.23–9.68% in terms of network latency, 3.40–13.66% in computational delay, and 1.03–11.55% in terms of system performance. Another real-world study in the healthcare domain [59], based on neural networks, achieved the lowest average turnaround time of 2.75 ms, lowest failure rate of 10%, and moderate CPU utilization of 60%, outperforming other algorithms in all metrics, thus making it highly efficient and reliable for the fog computing environment. Another relevant study in the healthcare domain based on fog computing [24] used RL as the base model, which demonstrated an improvement of 6.67% in average delay as compared to other existing models. Another study [71] used DRL in the fog computing healthcare domain, which demonstrated improved performance, reduced latency, increased accuracy, and higher confidence for correct predictions. The effective prediction and resource allocation method (EPRAM) [42] in the healthcare domain for fog environments resulted in improved makespan, average resource utilization, latency, and reduced energy consumption. Based on the evaluation of existing methods, DRL was the preferred learning algorithm as compared to other machine learning algorithms since it performed well in handling complex scenarios such as healthcare, smart cities, and industrial automation. This relevant study explored the use of ML to analyze and predict customer attrition in the banking sector, highlighting its significant impact on revenue and reputation. The key aspects include predictive modeling to identify customers at risk, tracking signs of dissatisfaction among customers, and comparative analysis of various ML techniques for effective churn prediction. A robust data preprocessing method ensures accuracy, while real-time monitoring through data visualization facilitates timely decision-making. Utilizing ML banks can create personalized offers, enhance customer services, and optimize resource allocation [90].

## 6. Conclusions

This study presents an in-depth SLR of ML-based resource management in fog computing using the PRISMA protocol. A thorough synthesis was carried out based on six research questions while considering 68 research articles based on the inclusion and exclusion criteria. The results show a prevalent trend toward utilizing ML and DL algorithms to address various challenges related to resource management in fog computing. This suggests a recognition of the potential of these algorithms in optimizing resource management, enhancing system efficiency, and improving performance. The analysis revealed that a wide range of ML/DL algorithms have been employed, including DRL, Q-learning, DQN, SVM, CNN, and RNN, which highlights that the complexity and diverse nature of fog computing challenges related to resource allocation and management need this diverse array of algorithms. There is a strong emphasis on addressing specific challenges, i.e., latency, resource allocation, energy consumption, and task offloading. The results revealed that these critical parameters need to be prioritized for the improvement of resource management in fog computing. Based on the analysis, it is concluded that the use of ML/DL techniques allows for handling complex and dynamic fog/IoT environments which require classification and prediction decisions in resource allocation. The focus on addressing latency, resource allocation, and energy consumption is consistent with the identified challenges. The adaptation of ML/DL techniques can lead to more efficient and optimized fog computing systems, reducing costs, and improving user experience and performance.

## Figures and Tables

**Table 1:** Comparison of current systematic literature review (SLR) with previous reviews.

ML	DL	NN	RL	Latency	Energy Consumption and Power Consumption	Resource Utilization	Cost Efficiency	QoS	Task Scheduling	Load Balancing and Service Placement	CPU Utilization	Scalability	Ref.
**✗**	✓	✗	✓	✓	✗	✗	✗	✗	✗	✗	✓	✗	[8]
**✓**	✓	✗	✓	✓	✓	✓	✓	✓	✓	✓	✓	✓	[9]
**✓**	✓	✗	✓	✓	✗	✗	✗	✗	✗	✗	✗	✗	[10]
**✓**	✓	✗	✗	✓	✓	✗	✗	✗	✗	✗	✗	✗	[11]
**✗**	✗	✗	✓	✗	✗	✓	✗	✗	✗	✗	✗	✗	[12]
**✓**	✗	✗	✗	✗	✗	✗	✗	✓	✗	✗	✗	✗	[13]
**✗**	✓	✗	✓	✗	✓	✗	✗	✗	✗	✓	✗	✗	[14]
**✓**	✗	✗	✗	✓	✗	✗	✗	✗	✗	✗	✗	✓	[15]
**✗**	✓	✗	✗	✓	✓	✗	✗	✗	✗	✓	✓	✗	[16]
**✗**	✓	✓	✓	✓	✓	✗	✗	✓	✗	✓	✗	✗	[17]
**✓**	✓	✓	✓	✓	✗	✗	✗	✓	✗	✓	✓	✓	[18]
**✗**	✗	✗	✗	✗	✗	✓	✗	✗	✗	✓	✗	✗	[19]
**✓**	✓	✓	✓	✓	✗	✗	✗	✗	✓	✓	✗	✗	[20]
**✓**	✓	✓	✓	✓	✓	✓	✓	✓	✓	✓	✓	✓	Proposed work

✓ Addressed, ✗ Not Addressed.

**Table 2:** RQs for SLR.

No.	Research Questions	Significance
**RQ 1.**	What are the state-of-the-art ML and DL techniques leveraging resource management in fog computing?	It will highlight the most recent and advanced ML/DL techniques applied in fog computing for resource management, which in turn will highlight potential opportunities for future research and development.
**RQ 2.**	What ML/DL algorithms are used for resource management in fog computing?	It will unravel the current capabilities, leading toward optimal resource allocation in fog computing.
**RQ 3.**	What are the significant challenges to optimizing resource management in fog computing using ML/DL?	It will underscore significant challenges faced in optimizing resource management in fog computing. Identification of robust ML/DL solutions for the issues will pave the way for more intelligent and efficient fog computing solutions.
**RQ 4.**	How can ML/DL enable dynamic resource allocation and distribution across the layers, i.e., edge–fog, edge–fog–cloud, or manage it on fog?	Imagine a system that can automatically allocate tasks and resources across all these different computing layers (edge, fog, and cloud) to achieve the best performance and save cost. Can ML help us build this super-smart system?

**Table 3:** Analysis of ML and DL algorithms for fog computing.

Cat **	Models	Usage/Application	Strengths	NOR *
**ML**	Decision trees	Optimal node selection	Straightforward, effective, and prone to overfitting with complex data	7
		Task distribution		
	KNN	Based on workload characteristics	Intuitive, adapts well to local variations, and can be computationally expensive	
		Adjusts load based on proximity		
	SVM	Optimal resource utilization	Task effective in complex scenarios, handling large datasets and nonlinear relationships	
	Random forest	Resource usage forecasting	Accurate, robust, and computationally intensive	
		Dynamic decision-making		
		Identifying idle resources		
	Naïve Bayes	Based on probabilistic predictions	Simple, efficient, fatigue in handling complex dependencies	
		Anomaly detection		
		Optimal assignment		
**FL**	Fuzzy logic (FL)	Uncertainty based modeling	Adapts well to uncertainty and requires expert knowledge for rule definition	4
	Neuro-fuzzy	Optimal resource policies	Integrates adaptive learning with robustness but is complex to implement and train	
**DRL**	Q-Learning	Adaptive resource allocation policies	Q-learning can adapt to dynamic environments. However, its convergence is slower	41
		Real-time decision-making		
	SARSA	Immediate action based on states	Effective for immediate rewards, weak response for delayed rewards	
		The decision is made based on learned rewards		
	Expected SARSA	Predicting rewards for allocations	Balances exploration and exploitation, is sensitive to reward estimation errors, and adjusts learning rates	
	AWRR	Real-time load balancing	It is effective for real-time load balancing, which requires continuous monitoring	
		** Allocating tasks to high-performance nodes		
	DQN	Optimizes resource allocation and task-offloading decisions	Effective in learning complex policies but computationally intensive	
	DDQN	Enhances Q-value estimation and stability in dynamic environments	Reduces overestimation bias, improving learning efficiency	
	DDPG	Manages continuous resource allocation and optimization	Handles complex tasks and constant action spaces effectively	
	DRQN	Manages tasks with temporal dependencies and dynamic environments	Suitable for tasks requiring memory of past states and actions	
	Advantage actor-critic (A2C)	Balances exploration and exploitation for adaptive resource management	Provides faster convergence and improved policy learning	
	Federated Deep Q-learning-based offloading (FedDOVe)	Enables collaborative optimization of resource allocation across distributed fog nodes	Enhances scalability and robustness in federated fog environments	
	Learning-in-the-fog (LiFo)	Enables autonomous decision-making at the edge for faster response times	Reduces latency and bandwidth consumption by minimizing reliance on centralized servers	
**DL**	CNN	Analyzes image or sensor data for real-time decision-making	Efficient for spatial data processing, suitable for image tasks in fog environments	13
	Graph convolution neural network (GCCN)	Manages network topology analysis, routing optimization, and graph-based resource allocation	Handles complex relationships in network data, improving scalability and performance	
	PNN	Predicts resource demand	Robust probabilistic modeling requires substantial training and computation.	
		Identifying deviation		

* Number of occurrences; ** categories/approaches.

**Table 4:** ML/DL algorithms used to address latency and relevant issues.

Technique	Explanation	Issues	Ref.
RL	Q-learning in RL for load balancing to optimize network performance.	Delay	[33]
Q-Learning			
Q-Network	Uses Q-networks and neural networks to improve handover efficiency and offloading decisions.		[36]
DRQN			
RRN			
DRRL			
MODRL	Applies multi-objective deep reinforcement learning (MODRL) to minimize task completion time and optimize resource utilization.	Minimize Latency	[29]
Q-Networks			
DQ-Learning	Reduces energy consumption at RSUs, balances computation load, and optimizes task offloading.		[30]
FedDOVe			
DRL	Deep Reinforcement Learning-based IoT application Scheduling algorithm (DRLIS) to reduce response time and balance server load.		[35]
BMNF	Utilizes NN for latency and energy consumption		[34]
DRL	Reduces delay, improves resource utilization, and optimizes costs.		[37]
DNN			
Multi-agent DRL	Uses multi-agent DRL to reduce end-to-end delay.		[38]
A2C	Applies DRL-based A2C algorithm for adaptive resource management.		[39]
DQ-Learning	Reduces energy consumption at RSUs, balances computation load, and optimizes task offloading.		[30]
FedDOVe			
A2C	Applies DRL-based A2C algorithm for adaptive resource management.		[40]
DDQN	Optimizes service placement and latency		
Decision Tree	Uses ML classifiers for optimizing latency and energy consumption.		[41]
DDQN	It uses DDQN and DQN phases to make offloading decisions and resource allocation.		
DRL	Utilizes DRL and NN for effective prediction and resource allocation (EPRAM).		
PNN			
MORL	It uses MORL and NN to optimize service placement in heterogeneous fog environments.		[42]
DNN			
DQN			
DQBRA	Uses DQN-based resource allocation (DQBRA) for optimal communication and computational delays.		[43]
DDN	Utilizes DL for joint offloading decisions and resource allocation in fog computing.		[44]
RNN	Improves handover efficiency and service continuity in the Internet of Vehicles (IoV) with intelligent fog node selection.		[45]
Feed-forward NN			
JODRA	DRL-based joint offloading decision and resource allocation (JODRA) with RL improves decision making Support vector regression (SVR) for workload prediction, reducing average delay and cost.		
RL			
SVR			
(F-AMLF)	Fuzzy-assisted machine learning framework (F-AMLF) optimize resource allocation reduce latency		[46]
GCCN	Enhances fog computing performance with intelligent resource allocation using GCCN.	Loop delay	[47]
AWRR	Advanced weighted round robin (AWRR)Uses Q-learning RL algorithms for optimizing makespan and resource allocation.	Makespan	[48]
RLFS	Optimizes task scheduling and reduces response time using RL and Fuzzy Systems (RLFS) techniques.	Response Time	[49]
CML	Utilizes collaborative ML (CML) in software-defined networking (SDN) CloudSimSDN v2.0 to optimize response time and energy consumption.		[24]
DQN	Minimizes waiting time, delay, and packet loss while maximizing task completion percentage.	Waiting Time	[50]
IRAT	End-to-end delay.		

**Table 5:** ML/DL algorithms address task offloading and its relevant issues.

Technique	Explanation	Issues	Refs.
DRL	It uses DRL and enhances versions to learn optimal offloading policies while considering resource availability, intelligent decisions, network conditions, and the nature of tasks.	Task Offloading	[35,46]
CDDN	Conditional deep neural networks (CDDN) are used for task offloading, where the tasks are efficiently placed on processing devices according to their needs.		[51]
CNN	This fog-enabled architecture using CNNs efficiently distributes processes between terminal fog and cloud layers.		[25,52]
ML	ML algorithms and classifiers (e.g., random forest, SVM) are used to predict the nature of the tasks and make informed offloading decisions.		[27,53,54]
FL and DQN	The combination of FL and DQN gives a novel task-offloading technique, which, overall, improves the efficiency of the system.		[47,55]
FL and neuro-fuzzy	A novel resource allocation model is presented by combining FL and neuro-fuzzy techniques. This hybrid approach uses FL to model the orchestration decision based on various factors, and the neuro-fuzzy model uses fuzzy sets and rules for decision-making.		[56]
NN DNN	It utilizes NN to learn complex patterns in data and optimize offloading decisions.		[43,57]
RNN	It uses RNN to predict resource requirements based on workload. In response, it dynamically adjusts the number of resources to match the workload.		[58]
PNN	This research work efficiently manages workload by employing probabilistic neural network (PNN)-based algorithms.		[59]
CNN	A CNN is used to predict the increasing volume of traffic patterns generated by IoT devices and enables proactive network management.		[60]
DRLIS	The DRLIS is used for adaptive decisions and is integrated with the FogBus2 framework to minimize execution cost, response time, and load imbalance, resulting in optimized resource utilization and efficient load balancing.	Offloading Decisions	[35]
DQN	DQN can learn optimal offloading decisions through interaction with the environment and rewards.		[26,61]
	It uses intelligent fog service placement and intelligent fog service schedular for task offloading.		[58]
DRL	It uses offloading as a strategy to optimize resource utilization by distributing the workload across multiple nodes. This approach addresses the challenges of a dynamic environment where task arrival rates and network conditions fluctuate, which affects offloading decisions.		[38]
DDQN	In this research, a novel placement algorithm based on DDQN is used, enabling optimal service placement in fog computing.		[40]
FL and DRL	A novel resource allocation model is presented by combining FL and neuro-fuzzy techniques. This hybrid approach uses FL to model the orchestration decision based on various factors, and the neuro-fuzzy model uses fuzzy sets and rules for decision-making.		[55,56]
FL and Q-learning	Uses fuzzy Q-learning approach for resource provisioning, efficiently allocating resources results in managing a dynamic workload.		[62]
DRL	It uses DRL for optimal task scheduling, considering task dependencies, resource availability, and network conditions.	Task Scheduling	[63,64]
DRL	DRL is used for efficient resource allocation, which is essential for supporting task migration and offloading decisions.	Task Migration	[44,59,65]
DRL and DQN	Offloads computation-intensive tasks to edge servers to reduce processing time.	Processing Time	[38,58]

**Table 7:** Overview of ML/DL algorithms used to address power consumption and its related issues.

Technique	Explanation	Issues Addressed	Refs.
Random forest, SVM, decision trees	ML algorithms, i.e., random forest, SVM, and decision trees, can predict the nature of tasks and their characteristics, which can make effective decisions about scheduling, can fairly manage resources, reduce idle time and overall power consumption, and lead to improved power efficiency.	Power efficiency	[27,28,54]
DRL, DDQN, Q-learning, DQN	DRL and RL algorithms are deployed to optimize task offloading, scheduling decisions, resource allocation, and resource utilization to minimize energy consumption by enhancing diverse policies that can handle workload variations and network conditions.	Energy consumption reduction	[26,30,35,37,38,40,44,45,53,65,68]
NN, CNN, RNN	Neural network architectures such as CNN, RNNs, and feed-forward networks optimize energy consumption by learning to predict resource demands and dynamically adjusting resource allocation based on workload characteristics, leading to improved energy efficiency.	Energy efficiency	[25,36,43,70,74]
FL, Neuro-fuzzy	Fuzzy systems can handle uncertainty, providing energy-efficient decision-making capabilities, and leveraging the system to adjust to real-time data and application for efficient resource allocation, which ensures optimal energy usage and reduces wastage in resource utilization.	Energy-efficient decision-making	[47,55,56,59,62]
DL, DNN, A2C	DL techniques optimize power consumption by allocating resources effectively, reducing idle time, and matching resource allocation to workload demands to minimize power usage.	Power Usage Minimization	[34,37,39,69,71]

**Table 8:** ML/DL algorithms used to address service placement and its related issues.

Technique	Explanation	Issues Addressed	Ref.
DQN	DQN for packet scheduling, which minimizes delay, enhances task scheduling, and fair resource allocation.	Minimize packet loss	[26]
		Waiting time	
		Task scheduling	
Random Forest	It uses ML with a K-fold random forest to address latency, which in turn improves QoS and system service performance.	Latency	[27]
		QoS enhancement	
Neural Framework	It uses DL neural frameworks to reduce latency, address energy consumption challenges, and find hospitals’ locations for services.	Energy consumption	[34]
		Locating fog node facility	
DQN	DRL is a power tool that supports adaptability, optimization, and autonomy. These DRL techniques, like DQN and Q-learning, are deployed for adaptive scheduling, load balancing, and optimizing weighted cost allocations, addressing latency issues effectively.	Adoptive scheduling	[35]
Q-learning		Load balancing	
		Weighted cost	
DRL	DDQN is deployed with priority experience replay for service placement in fog computing. It reduces service execution time and manages node energy. Priority experience replay enhances the learning rate and improves the system’s performance.	Service placement	[40]
		Service execution time	
		Node energy	
Fuzzy	Fuzzy-assisted ML techniques are applied for latency reduction, to lower energy consumption, and to improve decision-making for the placement of tasks on fog nodes.	Lower energy	[47]
ML		Cost-effective	
		Decision-making	
FL	Integrates neuro-fuzzy systems to manage the effective allocation of resources in fog computing.	Network latency	[56]
NN		Computational delay	
		Optimization resource management task offloading	
DRL	Deep Q-learning and RNN are applied to balance load, resource utilization, and energy consumption.	Load balancing	[57]
DQL		Resource utilization	
		Energy consumption	
		Response time	
A3C	Using A3C to manage dynamic workload, optimize resource utilization on fog servers, and meet deadlines.	QoS	[63]
Q-Networks		Service placement	

**Table 9:** Overview of ML/DL algorithms used to address cost and its related issues.

Technique	Explanation	Issues Addressed	Refs.
DQN Q-learning	DRL techniques such as DQN and Q-learning optimize resource allocation and load balancing to reduce the weighted costs associated with service operations.	Adoptive Scheduling Load Balancing Weighted Cost	[35]
DRL Q-Networks	Applies DRL and Q-networks to tackle QoS and service placement challenges while optimizing costs, ensuring that service deployments are cost-effective and aligned with performance metrics.	QoS Service Placement Problem Cost	[46]
Fuzzy ML Q-learning	Efficient resource utilization, minimizing energy consumption, and effective scheduling can reduce costs.	Cost-Effective Decision-making Efficiency	[47,62,76]

**Table 10:** Overview of ML/DL algorithms used to address load balancing and their related issues.

Technique	Explanation	Issues	Refs.
DQN	DQN helps prevent fog nodes either from overburdening or underutilization. IFSP provides a fair distribution of requests on fog nodes.	Load balancing	[26,68,78]
DRLIS	DRL addresses load balancing with the scheduling algorithm DRLIS. It optimizes the response time of heterogeneous IoT applications and balances the load of processing servers.	Load balancing	[35,61,79]
Federated DQL	A federated learning and DQL are used to balance the load across fog environments.	Load balancing	[30,57]
DL	Applies deep learning techniques to optimize energy efficiency load balancing balance and manage network usage effectively in fog computing scenarios.	Load balancing	[49]

**Table 11:** Overview of ML/DL algorithms used to address QoS and its related issues.

Technique	Explanation	Issue Addressed	Refs.
DRL	Uses DRL algorithms like DQL to optimize task offloading and resource allocation, reducing latency by making adaptive decisions based on real-time network conditions.	Latency	[35,40,44,61,71]
NN, ML	Neural frameworks such as CNNs and RNNs can be employed to predict and reduce latency by efficient task scheduling and resource utilization in network environments.		[34,36,43,70]
Q-Learning	Implements Q-learning in RL models to optimize load balancing and resource allocation, thereby enhancing throughput and system efficiency.	Throughput	[33,49,69]
DDQL	Enhances reliability and accuracy by using advanced RL techniques to minimize service delays, improve response times, and meet SLA requirements consistently.	Reliability and Accuracy	[53,81]
DNN	Integrates DNNs with RL/DRL methods to achieve high reliability and accuracy in task execution, ensuring precise decision-making under dynamic network conditions.		[42,82,83]
DQN	Utilizes DQN for optimal resource allocation and task scheduling to meet deadlines and prevent SLA violations, prioritizing critical tasks based on their requirements.	Deadline and SLA Violation	[26,30,84]
RL	Applies RL algorithms such as SARSA and Expected SARSA to enforce strict adherence to deadlines by optimizing task completion times and reducing waiting periods.		[29,31,68,85]

**Table 12:** ML/DL algorithms used to address challenges in fog computing environments.

ML/DL	Algorithms	Key Impacts	Issue Addressed
ML	Decision Tree, SVM, PNN, random forest, LR, naïve Bais, ML	Resource optimization, energy management, decision-making	Latency, Energy Consumption
RL	DRL, DRLIS, DDQN, DQ-Learning, A2C, RLFS, DRL-based IoT Scheduling, multi-agent DRL	Dynamic decision-making, latency reduction, resource optimization, energy efficiency, real-time performance, responsiveness	Latency, Resource Optimization, Energy Consumption, Response Time
DL	BMNF, FedDOVe, MORL, F-AMLF, Collaborative ML, DQBRA, DDN, DQN	Complex pattern recognition, performance improvement, response time optimization, Federated learning, multi-objective optimization, collaborative learning, computational efficiency, accuracy, reliability, improved user experience, system performance	Energy Consumption, Performance Improvement, Resource Optimization, Response Time

**Table 13:** A categorization of key challenges and potential solutions for fog computing.

Cat *	Challenges	Challenge Description	IRM **	Solution Prospect	IA ***
Computational Resource and Latency	Limited Resources	Processing Power	Complex models undeployable	Resource-efficient algorithms	DL Algorithms (e.g., CNN, RNN)
		Memory		Efficient model training techniques	
		Storage		Lightweight models	
				Compressed models	
	Latency Constraints	Critical Factor for real-time applications	Miss time-critical task	Prioritize time-bond tasks to meet the deadline	DRL
		Network delay can impact performance			
	Energy Consumption	Balancing performance and energy-efficient	Increase operation cost	Optimizes resource allocation for critical tasks while considering energy efficiency	SVM
			Reduce device life span		
Scalability and Management	Scalability	Dynamic workload	System complexity	Auto Scaling	ML/DL
		Large-scale fog node management	Resource Provisioning	Distributed resource management	
	Deployment and maintenance	Complex deployment due to node heterogeneity	Monitoring	Resource allocation	ML/DL (DQN, DDQN, DRL, MORL, ML)
		Infrastructure	Fault Tolerance	Task offloading	
				Enhance QoS	
	Resource Allocation	Dynamic workload	Cost-effectiveness	Task Scheduling	(ML, DRL, NN, FNN, DQN)
		Resource heterogeneity	User experience	Resource provisioning	
		Latency constraints	System performance		
Data Availability and Quality	Limited Data	Insufficient historical data is available for training	Inaccurate Model	Generate synthetic data	
			Performance inefficiency	Transfer learning	
				Data augmentation techniques	
	Data Heterogeneity	Data Processing	Inefficient resource utilization	Effective data management	
		Data integration	·Model Complexity	Robust ML/DL techniques used	
	Data Privacy and Security	Inconsistency format Difficult Data Aggregation	Computation overhead Storage requirement	Federated learning	
Model Complexity and Interpretability	Model Complexity	Computation overhead	Potential performance issues	Model optimization	ML/DL
		Overburden		Efficient resource utilization	
	Model Explainability	Trust and reliability	decision-making	Recognize patterns	ML/DL
	Model Adaptability	Adopt network conditions	Deployment efficiency	Learning Models	DL
				Value updating	

* Categorization of challenges; ** impact on resource management; *** impact algorithm.

**Table 14:** Overview of algorithms for resource management across edge, fog, and cloud layers.

Algorithms	Architecture	Task Services	Ref.
DQN	IoT–Fog–Cloud	Tasks are managed across all layers	[26]
SVM	IoT–Fog–Cloud	Tasks served across IoT, fog, and cloud layers	[27]
SVM	Fog–Cloud	Tasks managed across fog and cloud	[28]
DQN	Mist–Fog–Cloud	Tasks managed in mist, fog, and cloud	[33]
DRL	Edge–Fog	Edge-to-fog task migration based on conditions	[35]
DRL	Fog–Cloud	Tasks handled in fog, with potential cloud migration	[38]
DNN	Fog–Cloud	Tasks executed primarily in fog with cloud capabilities	[43]
RLFS	Edge–Fog–Cloud	Tasks are scheduled dynamically across edge, fog, and cloud	[50]
Fuzzy Logic	IoT–Fog–Cloud	Tasks are managed with FL across all layers	[55]
Neuro-Fuzzy	Edge–Fog–Cloud	Tasks managed across edge, fog, and cloud with neuro-fuzzy techniques	[56]
CNN	Fog–Cloud	Tasks processed on fog and cloud	[61]
DNN	Fog–Cloud	Tasks served in fog and possible cloud migration	[88]
DNN	Edge–Fog	Tasks managed across edge and fog	[89]

**Table 15:** Practical implementation of ML/DL algorithms.

Real-Time Scenarios	Algorithms Used	Key Metrics	Validation	Ref.
Industrial Monitoring and Analysis	CNN	Energy, Production efficiency	The fog-enabled system improved energy efficiency and production output, reduced bandwidth and data transfer time, and allowed for faster fog layer processing over the cloud. This improvement was validated in a real manufacturing environment.	[25]
Smart Cities Traffic Control	RL	Smart Cities or IoT environments for Fog Computing resource management	Using a single payload and varying traffic in a no-deadline scenario, with deadlines and two payloads, and using real-world traffic distributions, the SARSA algorithm maximized task completion across all of these. The performance was further supported by each node acting as an independent learner and its consistently superior performance in maximizing the rate of tasks completed within deadlines.	[88]
Vehicular fog networks	DRL	Energy Consumption, Latency, Task Completion Rate, Load Balancing	This system addresses energy concerns, minimizes latency, and optimizes resource use, leading to performance benefits for vehicular fog computing and practical applications in smart transportation systems.	[30]
	Neural Network	Latency	Validation was conducted through experiments that compared predicted costs against actual values, confirming the models’ effectiveness in minimizing service interruptions during handovers.	[36]
	DRL	Task Offloading, System Cost, End-to-End Delay, Processing Time	The Proposed Federated Multi-Agent DRL algorithm showed significant improvements in task residence time, end-to-end delay, and overall system cost.	[38]
	DRL	Miscellaneous User Requests	Maximizes satisfied user requests within a delay threshold and outperforms heuristic approaches.	[78]
	DRL	Mean Utility, Completion Ratio	These key results highlight the effectiveness of the proposed SBPO algorithm in real-time vehicular environments, its superior performance compared to existing methods, and the thorough validation process that supports its efficacy.	[83]
Healthcare	RL	Makespan, Completion Time, Dynamic Resource Allocation	Testbed deployment, performance evaluation, robustness and adaptability	[24]
	DRL	Latency, Task Assignment, Resource Utilization	Comparison with state-of-the-art load balancing algorithms (LC, RR, WRR, AWRR)	[42]
	Neuro-Fuzzy	Network Latency, Computational Delay, Optimization, Resource Management, Task Offloading	Extensive simulations demonstrating efficacy in network, computing, and system performance.	[56]
	Neural Networks	Load Balancing, Latency, Task Migration, Failure Rate, Task Priority, QoS	ELBS offers a promising approach to load balancing in FC environments for healthcare applications. It effectively addresses key challenges like resource allocation, task scheduling, and data management. However, further research is needed to evaluate its performance in real-world scenarios and to address potential scalability issues.	[59]
	RNN	Latency, Resource Utilization, Energy Efficiency	FogDLearner should incorporate dynamic scalability to analyze the execution time scale with the number of devices.	[70]
	DRL	Resource Consumption, Power Consumption, Network Bandwidth, Latency, Accuracy	Real-world healthcare data, clinical trials	[71]
	DR Transformer Model	--	The real-time predictive capabilities of the Transformer model allow for the accurate assessment of walking patterns and provide tailored recommendations for ankle–foot orthosis (AFO) usage. A feedback loop through physician validation and patient monitoring ensures an adaptive treatment plan, thus validating the potential for real-time clinical use.	[91]

## Data Availability

No new data were created or analyzed in this study.
